# The shootings in Oslo and Utøya island July 22, 2011: Lessons for the International EMS community

**DOI:** 10.1186/1757-7241-20-4

**Published:** 2012-01-26

**Authors:** David J Lockey

**Affiliations:** 1 Consultant, North Bristol NHS Trust & London's Air Ambulance, Royal London Hospital, London E1 1BB; 2 Hon. Professor, Trauma & Pre-hospital Emergency Medicine, School of Clinical Sciences, University of Bristol, UK

## 

SJTREM has published an account by Sollid and colleagues of the pre-hospital medical response to the major incidents, which occurred in Oslo and Utøya island on July 22, 2011 [1]. Although very similar incidents have occurred in Europe and elsewhere, this terrible day saw the greatest loss of life recorded in this type of incident in recent times. Internationally EMS providers looked on with the certain knowledge that this type of incident is sadly one that we all have to prepare for. It is unrelated to national foreign policy, religious extremism or the existence of known terrorist activity. In short this type of incident is unpredictable and has the potential to happen in any community at any time.

The aftermath of this type of incident is complex. Initially public and private grief dominates. There is then an attempt to comprehend the motives and background to apparently senseless acts that led to the tragic loss of innocent lives. Usually there is intense scrutiny of the perpetrator in an attempt to identify something in his or her background which might have predicted the incident. Inevitably the emergency response to the incidents is examined sometimes objectively and sometimes less so. The families of the victims and the public want to be reassured that everything possible was done to prevent the loss of life. Government legislation may result from incidents - the Dunblane school killings in Scotland in 1996 which resulted in the death of fifteen small children and their teacher led to rigorous controls on gun ownership in the UK. Later the emergency services can be subject to further scrutiny at inquests or public inquiries. These can be months or years after the event (the inquests into the London bombings in 2005 were only completed five years later). The motivations behind requests for information about the emergency response are varied. However, placing objective information about the emergency service response in the public domain is a responsible act. It allows other EMS services nationally and internationally to analyse and exercise their emergency responses to similar incidents with credible background information. This may allow improved emergency responses to future incidents which may occur before the conclusions of lengthy public or legal investigations. The authors of this paper are to be commended for bringing objective information into the medical literature in a timely manner after managing a difficult incident with great skill.

Although all major incidents are unique they all have common elements. The incidents in July in Norway can be usefully categorised to analyse the challenges faced by the regional EMS system.

## 'Lone Wolf' incidents

These incidents were difficult to predict. They are categorised by some authorities as 'lone wolf' incidents - usually planned and executed by one individual and unconnected to known organisations. They are often carefully planned. Other well documented 'lone wolf type incidents' in the US include the Oklahoma bombing by Timothy McVeigh in 1998 (168 deaths) [2] and the long letter bomb campaign of Theodore Kaczynski. In Europe the apparently racially motivated killings by John Ausonius in Sweden and Franz Fuchs in Austria in the early 1990s, two school shooting incidents in Finland in 2007 and 2008, and a series of nail bombs in London by David Copeland in 1999 are all examples of this type of incident. Although in retrospect, many of the perpetrators of these incidents have exhibited dysfunctional behaviour, intelligence on them or their intentions is rarely available since they are not usually members of organisations.

## Multiple incidents

Two incidents are described a few hours apart. Where more than one major incident occurs simultaneously coordination and allocation of adequate resources is always more difficult. There are numerous recent terrorist incidents that have involved multiple sites: the Istanbul bombings in 2003, the Madrid bombings in 2004 [3], the London bombings in 2005 [4] and, most notably, the Mumbai shootings in 2008 where ten incidents occurred in one city. Despite this challenge the response times described in this article are excellent. In Oslo the first ambulance was on scene three minutes after the first emergency call and a major incident was declared at eight minutes. At twenty six minutes there were 41 ambulances on scene and at 90 minutes all immediate needs had been met and the emergency resources were effectively ready for redeployment. Examination of the available literature reveals that most advanced EMS systems would struggle to achieve these timelines. At Utøya island the challenges were more complex. In a more rural location the first ambulance was on scene in nine minutes and a major incident declared at 21 minutes. Victims could not be attended for more than an hour but the complex scene was cleared of victims two hours after first attendance. Despite the non-urban location of the incident a considerable number of ambulances and air ambulances with physicians were able to quickly attend and evacuate the victims after access was achieved.

## Scene safety

The problem of an armed perpetrator on scene preventing access to victims is a relatively common problem in so called 'spree killing' incidents. The perpetrators often commit suicide but even when this happens confirmation of scene safety often takes time. A comprehensive armed police response is almost always more rapid in urban areas since this is where most incidents occur. A slower response in more rural locations is almost inevitable. In 2010 twelve people were killed by a gunman in Cumbria, a rural county in the UK. The gunman was mobile and was able to operate at multiple locations for more than two hours before shooting himself. The scene at Utøya island was additionally complicated by the presence of a powerful weapon with long range and separation of the scene from the mainland by water. In addition to rescuer access the water made self evacuation from the scene difficult. The previously described concept of 'reverse triage'[5] - where the least injured patients present to medical rescuers before the more seriously injured immobile patients is described here. This appears to have been predicted and managed well. Other scene hazards that are described include bad weather and the presence of twelve helicopters operating in the area.

## Lessons learned

The authors describe a number of potential lessons that emerged after early analysis of the incident. They describe some failure of communication. It is unclear whether this had any significant impact on patient outcome. It is important to note that no well reported major incident has ever been free of communication issues. Providing good care without perfect communication should be the aim of all EMS systems. This appears to have happened in these incidents and may have been due to the presence of senior physicians and paramedics at the scene. Analysis of multiple scenes after the London bombings suggested improved triage and low mortality associated with physician-paramedic teams on scene [5]. This was the model used at these incidents and the mortality after attendance by physician-paramedic teams is reported as very low. The fact that sixty flight movements were recorded during this incident does demonstrate the importance of air ambulances at this type of incident. It would be valuable to model the likely attendance times of advanced medical teams and evacuation times of casualties without the use of helicopters. The need to re-site the casualty clearing station is a good learning point but the initial site may well have appeared reasonable given the information available at the time. Security after identification of the threat appears to have been excellent. The authors suggest that a national triage guideline is required. It is difficult to know how many high level practitioners really rely on triage tools at major incidents but having one recognised system would seem good practice.

This paper provides a valuable insight into a complex and unpredictable incident. Other EMS systems may well benefit from the initial lessons learned. Similar incidents on a smaller scale are reported regularly and have been reportedly increasing in frequency [6]. This report also highlights the need for an international template to identify the medical lessons learned from major incidents which can be produced easily and quickly to propagate widely to the international EMS community. The pre-hospital response described in this article is impressive and it is difficult to see how the mortality in these incidents could have been improved. While the scale of this tragedy is immense for Norway, many aspects of the response to it will be viewed by international EMS systems as an example of good practice in major incident management.
